# Management of blunt extracranial traumatic cerebrovascular injury: a multidisciplinary survey of current practice

**DOI:** 10.1186/1749-7922-6-11

**Published:** 2011-04-08

**Authors:** Mark R Harrigan, Jordan A Weinberg, Ya-Sin Peaks, Steven M Taylor, Luis P Cava, Joshua Richman, Beverly C Walters

**Affiliations:** 1 Division of Neurosurgery, University of Alabama, Birmingham, Birmingham, Alabama, USA; 2 Division of Trauma and Critical Care Surgery, University of Tennessee Health Science Center, Memphis, Tennessee, USA; 3 University of Alabama, Birmingham School of Medicine, Birmingham, Alabama, USA; 4 Division of Vascular Surgery, University of Alabama, Birmingham, Birmingham, Alabama, USA; 5 Department of Neurology, University of Alabama, Birmingham, Birmingham, Alabama, USA; 6 Division of Preventative Medicine, University of Alabama, Birmingham, Birmingham, Alabama, USA

## Abstract

**Background:**

Extracranial traumatic cerebrovascular injury (TCVI) is present in 1-3% of all blunt force trauma patients. Although options for the management of patients with these lesions include anticoagulation, antiplatelet agents, and endovascular treatment, the optimal management strategy for patients with these lesions is not yet established.

**Objective:**

Multidisciplinary survey of clinicians about current management of TCVI.

**Methods:**

A six-item multiple-choice survey was sent by electronic mail to a total of 11,784 neurosurgeons, trauma surgeons, stroke neurologists, and interventional radiologists. The survey included questions about their choice of imaging, medical management, and the use of endovascular techniques. Survey responses were analyzed according to stated specialty.

**Results:**

Seven hundred eighty-five (6.7%) responses were received. Overall, a total of 325 (42.8%) respondents favored anticoagulation (heparin and/or warfarin), 247 (32.5%) favored antiplatelet drugs, 130 (17.1%) preferred both anticoagulation and antiplatelet drugs, and 57 (7.5%) preferred stenting and/or embolization. Anticoagulation was the most commonly preferred treatment among vascular surgeons (56.9%), neurologists (50.2%) and neurosurgeons (40.7%), whereas antiplatelet agents were the most common preferred treatment among trauma surgeons (41.5%). Overall, 158 (20.7%) of respondents recommended treatment of asymptomatic dissections and traumatic aneurysms, 211 (27.7%) did not recommend it, and 39.4% recommended endovascular treatment only if there is worsening of the lesion on follow-up imaging.

**Conclusions:**

These data demonstrate the wide variability of physicians' management of traumatic cerebrovascular injury, both on an individual basis, and between specialties. These findings underscore the need for multicenter, randomized trials in this field.

## Background

Blunt extracranial traumatic cerebrovascular injury (TCVI) is found in some 1-3% of all blunt force trauma patients [1-15]. Estimates of overall neurological morbidity associated with TCVI range as high as 31% [2,14,16]. Ischemic stroke appears to be the greatest source of neurological morbidity in this setting. A recent report of 147 patients with TCVI found an ischemic stroke rate of 12% attributable to carotid injuries and 8% due to vertebral artery injuries [2]. Although antithrombotic therapy to prevent ischemic stroke has been widely reported, several different options exist, including anticoagulation[2,7,9,17-19] and antiplatelet therapy [2,16,20-22]. Furthermore, the use of endovascular techniques in patients with TCVI appears to be gaining in popularity [23-26].

The optimal management strategy for patients with TCVI has not yet been established. No randomized trials in the management of patients with TCVI have yet been published. The issue is complicated by the complex nature of many patients with TCVI, such as the variety of cerebrovascular injuries as well as the presence of polytrauma. Furthermore, cerebrovascular injury in trauma patients frequently involves the participation of numerous specialists, such as neurosurgeons, trauma surgeons, stroke neurologists, and interventional neuroradiologists. Differing disciplines may have different perspectives and practices in the management of patients with TCVI.

The purpose of the current investigation was to assess the current management of patients with TCVI across the United States and also across the various medical specialties involved with the management of patients with TCVI.

## Methods

A six-item multiple-choice survey was sent by electronic mail to 11,784 members of the American Association for the Surgery of Trauma, the American Association of Neurological Surgeons, the American Heart Association Stroke Council, and the Society for Clinical Vascular Surgery. Email addresses were obtained from published membership lists. The authors attempted to exclude email addresses that overlapped between organizations. This project was approved by the Institutional Review Board.

Results were collected on a commercial survey website (http://www.surveymonkey.com). Only a single mass emailing was completed, and the survey was closed after one month. No follow-up emails or repeat email solicitations were used. All responses were kept completely confidential.

Standard two-sided chi-square tests were used to test for significant associations between specialty and survey responses. Because some expected cell counts were less than 5, results were confirmed using Monte-Carlo approximations of Fisher's exact test with one million repetitions. Testing was done using R version 2.10.1.

## Results

A total of 785 responses were received, representing an overall response rate of 6.7%. Members of the American Association for the Surgery of Trauma had the highest response rate, at 15.7% (Table 1). Several emails were received from recipients of the survey, explaining that they were not clinicians, not physicians, or did not take care of patients with TCVI.

**Table 1:** Responses according to professional society

	Number of survey requests sent	Number of responses
American Association of Neurological Surgeons	5,481	335 (6.1%)
American Association for the Surgery of Trauma	923	145 (15.7%)
American Heart Association Stroke Council	4,638	263 (5.7%)
Society for Clinical Vascular Surgery	742	42 (5.7%)

### Overall survey results

The total responses to the survey questions are listed in Table 2. The largest number of respondents were neurosurgeons (342, 45.2%) and the next largest responding specialty was neurology (205, 27.1%). Only 46 of the respondents (6.0%) reported seeing no TCVI cases each year; the most common frequency was 1-5 per year, which was reported by 442 (57.4%) of the respondents. A conservative estimate of the total number of TCVI cases seen by the respondents can be estimated by multiplying number of respondents reporting each range of cases per year by the lowest number in each range. Thus, as a group, the respondents estimated that they see at least 2,680 TCVI cases each year.

**Table 2:** Overall responses to the questionnaire

1.	What is your specialty?
	• Trauma surgeon = 137 (18.1%)
	• General surgeon = 19 (2.5%)
	• Neurosurgeon = 342 (45.2%)
	• Vascular surgeon = 52 (6.9%)
	• Neurologist = 205 (27.1%)
	• Interventional radiologist = 30 (4.0%)

2.	What is the approximate number of traumatic carotid or vertebral artery dissections or other injuries that you see per year?

	• None = 46 (6.0%)
	• 1-5 = 442 (57.4%)
	• 5-10 = 144 (18.7%)
	• > 10 = 138 (17.9%)

3.	What is your preferred method of imaging?

	• MRI/MRA = 175 (22.8%)
	• CTA = 464 (60.5%)
	• Doppler = 13 (1.7%)
	• Catheter angiography = 115 (15.0%)

4.	In most cases which treatment do you prefer?

	• Anticoagulation (heparin and/or warfarin) = 325 (42.8%)
	• Antiplatelet drugs = 247 (32.5%)
	• Both anticoagulation and antiplatelet drugs = 130 (17.1%)
	• Stent and/or embolization = 57 (7.5%)

5.	How would you manage a patient with intraluminal thrombus and no related neurological symptoms?

	• Thrombolytics = 47 (6.2%)
	• Heparin and/or warfarin = 500 (65.7%)
	• Antiplatelet drugs = 174 (22.9%)
	• None of the above = 40 (5.3%)

6.	Should asymptomatic traumatic dissections and traumatic aneurysms be treated with endovascular techniques, such as stenting and/or embolization?

	• Yes = 158 (20.7%)
	• No = 211 (27.7%)
	• Only if there is worsening of the lesion on follow-up imaging = 394 (51.6%)

The most common preferred method of imaging was computed tomographic angiography (CTA, 22.8%), followed by MRI/MRA (22.8%) and catheter angiography (15.0%). The most common preferred treatment was anticoagulation (42.8%) and antiplatelet drugs (32.5%). Regarding management of a patient with intraluminal thrombus and no related symptoms, the most common choice was heparin and/or warfarin (65.7%), followed by antiplatelet drugs (22.9%) and thrombolytics (6.2%). Some 20.7% of the respondents recommend treatment of asymptomatic dissections and traumatic aneurysms with endovascular techniques, while 2.7% would not and 51.6% would do so only if there were worsening of the lesion on follow-up imaging.

### Analysis by specialty

For each question there was a statistically significant association between response and medical specialty (all *P *< 0.00005 for both chi-square test and Fisher's exact test). The medical specialties with the greatest annual number of TCVI cases seen per respondent were interventional radiologists, followed by trauma surgeons and neurologists (Table 3). Regarding imaging, CTA was favored by a majority of respondents in each specialty, although 39.0% of neurologists preferred MRI/MRA (Table 4). Some 26.7% of interventional radiologists and 21.8% of neurosurgeons preferred catheter angiography. Anticoagulation was the most common preferred treatment among neurosurgeons, vascular surgeons, and neurologists, whereas antiplatelet agents were most commonly favored among trauma surgeons and general surgeons (Table 5). A minority of respondents in each specialty, ranging from 3.0% to 10.7%, preferred stenting and/or embolization. Responses to questions about treatment of asymptomatic lesions are listed in Table 6. For patients with an asymptomatic intraluminal thrombus, the majority of respondents in all specialties preferred heparin and/or warfarin; antiplatelet agents were the next most commonly favored treatment, followed by thrombolytics. Regarding asymptomatic dissections and traumatic aneurysms, the most common opinion among all specialties was that endovascular techniques should either not be used or they should be reserved for lesions that are found to worsen on follow-up imaging. However, neurosurgeons, trauma surgeons, and general surgeons were significantly more likely than vascular surgeons, neurologists and interventional radiologists to recommend endovascular treatment of asymptomatic lesions.

**Table 3:** Case volume by specialty

Question: What is the approximate number of traumatic carotid or vertebral artery dissections or other injuries that you see per year?				
	**None**	**1 to 5**	**5 to 10**	**> 10**

Neurosurgeon n = 342	28 (8.2%)	237 (69.5%)	35 (10.3%)	41 (12.0%)
Trauma surgeon n = 136	2 (1.5%)	58 (42.6%)	29 (21.3%)	47 (34.6%)
General surgeon n = 19	4 (21.1%)	6 (31.6%)	4 (21.1%)	5 (26.3%)
Vascular surgeon n = 52	4 (7.7%)	36 (69.2%)	9 (17.3%)	3 (5.8%)
Neurologist n = 204	6 (2.9%)	102 (50.0%)	61 (29.9%)	35 (17.2%)
Interventional radiologist n = 30	0	6 (20.0%)	8 (26.7%)	16 (53.3%)

**Table 4:** Preferred imaging by specialty

Question: What is your preferred method of imaging?				
	**MRI/MRA**	**CTA**	**Doppler**	**Catheter angiography**

Neurosurgeon n = 339	72 (21.1%)	189 (55.8%)	4 (1.2%)	74 (21.8%)
Trauma surgeon n = 137	6 (4.4%)	127 (92.7%)	0	4 (2.9%)
General surgeon n = 19	6 (31.6%)	12 (63.2%)	0	1 (5.3%)
Vascular surgeon n = 52	7 (13.5%)	40 (76.9%)	3 (5.8%)	2 (3.8%)
Neurologist n = 205	80 (39.0%)	87 (42.4%)	6 (2.9%)	32 (15.6%)
Interventional radiologist n = 30	2 (6.7%)	20 (66.7%)	0	8 (26.7%)

**Table 5:** Preferred treatment by specialty

Question: In most cases which treatment do you prefer?				
	**Anticoagulation**	**Antiplatelet drugs**	**Both**	**Stent/embolization**

Neurosurgeon n = 337	137 (40.7%)	105 (31.2%)	59 (17.5%)	36 (10.7%)
Trauma surgeon n = 135	39 (28.9%)	56 (41.5%)	34 (25.2%)	6 (4.4%)
General surgeon n = 19	7 (36.8%)	8 (42.1%)	2 (10.5%)	2 (10.5%)
Vascular surgeon n = 51	29 (56.9%)	8 (15.7%)	9 (17.6%)	5 (9.8%)
Neurologist n = 202	101 (50.0%)	71 (35.1%)	24 (11.9%)	6 (3.0%)
Interventional radiologist n = 30	13 (43.3%)	13 (43.3%)	2 (6.7%)	2 (6.7%)

**Table 6:** Management of asymptomatic lesions by specialty

**Question: How would you manage a patient with intraluminal thrombus and no related neurological symptoms?**				
	Thrombolytics	Heparin and/or warfarin	Antiplatelets	None of the above
Neurosurgeon n = 339	35 (10.3%)	205 (60.5%)	85 (25.1%)	14 (4.1%)
Trauma surgeon n = 135	7 (5.2%)	82 (60.7%)	34 (25.2%)	12 (8.9%)
General surgeon n = 19	2 (10.5%)	12 (63.2%)	3 (15.8%)	2 (10.5%)
Vascular surgeon n = 52	2 (3.8%)	39 (75.0%)	4 (7.7%)	7 (13.5%)
Neurologist n = 202	1 (0.5%)	148 (73.3%)	46 (22.8%)	7 (3.5%)
Interventional radiologist n = 29	0	22 (75.9%)	6 (20.7%)	1 (3.4%)
**Question: Should asymptomatic traumatic dissections and traumatic aneurysms be treated with endovascular techniques, such as stenting and/or embolization?**				
	Yes	No	Only if there is worsening on follow-up imaging	
Neurosurgeon n = 339	85 (25.1%)	66 (19.5%)	188 (55.5%)	
Trauma surgeon n = 134	37 (27.6%)	33 (24.6%)	64 (47.8%)	
General surgeon n = 19	5 (26.3%)	7 (36.8%)	7 (36.8%)	
Vascular surgeon n = 52	8 (15.4%)	20 (38.5%)	24 (46.2%)	
Neurologist n = 202	25 (12.4%)	86 (42.6%)	91 (45.0%)	
Interventional radiologist n = 30	4 (13.3%)	7 (23.3%)	19 (63.3%)	

## Discussion

The overall response rate in this study, 6.7%, is lower than the response rates reported in other published neurosurgical and trauma email surveys, which have ranged from 11.4% to 56% [27-31]. However, a significant number of the recipients of this email survey were either not clinicians or are clinicians who do not see patients with TCVI. The authors received several emails from recipients of the survey explaining this. For instance, many members of the AANS are neurosurgeons who do not see trauma patients, and a number of members of the AHA Stroke Council are Ph.D.s or nurses who also do not participate in the care of patients with traumatic injury. Furthermore, the recipients of the survey who did respond may account for a significant percentage of the clinicians who actually do take care of patients with TCVI in the United States. The lowest estimated total number of TCVI cases per year seen by the respondents is 2,680. The average annual number of blunt trauma admissions from 2000 to 2004 in the United States, as tabulated by the National Trauma Data Bank, was 162,306 [32]. Therefore, the lowest estimate of TCVI cases seen annually by the survey respondents represent approximately 1.7% of the total number of blunt trauma admissions in the United States, which is within the range of the overall incidence of TCVI (1-3%) among blunt trauma patients [1-15]. Thus, despite the seemingly low survey response rate, the respondents of this survey may represent a sizable fraction of the clinicians managing TCVI in the United States.

This survey demonstrates considerable variability in all aspects of the management of patients with TCVI, from imaging to medical therapy and the use of endovascular techniques. The most commonly preferred method of imaging was CTA, which likely reflects the ubiquity of CT scanning in the work-up of trauma patients, the widespread use of CTA for screening of trauma patients who are at risk of having a TCVI, and numerous published studies of CTA in this setting [14,33-37]. However, a significant subset of respondents (22.8%) favored MRI/MRA. This modality was most popular among neurologists, of whom 39.0% favored MRI/MRA. This may reflect current practice in the management of patients with spontaneous cervical artery dissection as expressed in a recent survey of members of the British Association of Stroke Physicians, 90% of whom indicated MRI/MRA as their preferred method of imaging in that setting. Overall, only 15% in the present survey preferred catheter angiography. Recently published guidelines for the management of blunt cerebrovascular injury by the Eastern Association for the Surgery of Trauma concluded that four-vessel cerebral angiography remains the gold standard for diagnosis, that duplex ultrasonography is not adequate for screening, and that multislice (eight or greater) CTA may be considered as a screening modality in place of catheter angiography[38] The authors of the guidelines also recommended that follow-up catheter angiography be done for grades I to III injuries. Grade I injuries include intimal irregularities with < 25% narrowing; grade II injuries consist of dissections or intramural hematomas with > 25% narrowing; and grade III injuries are dissecting aneurysms [39].

With respect to management, the most commonly preferred treatments overall were anticoagulation (42.8%) and antiplatelet agents (32.5%). These results are virtually identical to the findings of the British survey about spontaneous cervical artery dissection; those respondents were also divided between preferring anticoagulation (50%) or antiplatelet agents (30%) [40]. A number of studies of TCVI have found an association between antithrombotic therapy and lower ischemic stroke rates [2,7,9,14,17-19,41], although a cause and effect relationship has not been demonstrated in a controlled study. Treatment of patients with TCVI with anticoagulation using heparin and warfarin has been more widely reported than treatment with antiplatelet agents [2,7,9,17-19]. However, systemic anticoagulation is associated with bleeding complication rates up to 16% [7,14,17,42] and up to 36% of patients with TCVI are not candidates for systemic anticoagulation due to coexistent injuries [2,20]. Antiplatelet therapy (single agent treatment with aspirin is the most commonly reported regimen) may have a lower risk of complications and several retrospective studies have indicated that antiplatelet therapy is equal to or superior to anticoagulation in terms of neurological outcomes [2,16,20-22].

The Eastern Association for the Surgery of Trauma blunt TCVI guidelines made treatment recommendations according to the type of lesion [38]. Barring contraindications, antithrombotic medications such as aspirin or heparin were recommended for grade I and II TCVIs. The authors of the guidelines concluded that either heparin or antiplatelet therapy may be used with seemingly equivalent results. Although they stated that they could not make any recommendations about how long antithrombotic therapy should be administered for patients receiving anticoagulation, the authors recommended treatment with warfarin for 3 to 6 months. They recommended consideration of surgery or endovascular treatment of grade III lesions (dissecting aneurysms), and surgical or endovascular repair of carotid lesions associated with an early neurological deficit.

Regarding the management of asymptomatic lesions, the majority of respondents overall (65.7%) would manage a patient with a clinically silent intraluminal thrombus with heparin and/or warfarin, whereas 22.9% would use antiplatelet drugs and 6.2% would use thrombolytics. Additionally, 20.7% would use stenting and/or embolization to treat asymptomatic dissections and traumatic aneurysms, while a slim majority (51.6%) would use these techniques only if there were worsening of the lesion on follow-up imaging. The question of the management of asymptomatic TCVI lesions is important because of the widespread use of CTA screening protocols. Screening protocols call for CTA imaging of blunt trauma patients with risk factors for TCVI, such as cervical spine injuries and skull base fractures. Screening of asymptomatic patients is somewhat controversial [38], as some data indicates that a significant number of ischemic strokes due to TCVI occur prior to diagnosis [2,43], and that asymptomatic TCVI lesions may carry a relatively low risk of subsequent stroke, particularly when some variety of antithrombotic therapy is used. Thus, the situation with extracranial TCVI may be analogous to extracranial atherosclerotic disease, in that asymptomatic lesions carry a much more benign prognosis than symptomatic lesions. Differentiation in outcomes and management options between symptomatic and asymptomatic TCVI lesions is fertile ground for future investigation.

Endovascular treatment with stenting and/or embolization was the preferred method of treatment for 7.5% of the respondents overall, and was most popular among neurosurgeons (10.7%), compared to other specialists. The use of endovascular techniques in the management of patients with TCVI has been reported with increasing frequency in recent years [16,23-26,44-49]. However, compared to the other issues surrounding TCVI, the actual clinical benefit of endovascular treatment remains the least well defined, underscoring the need for prospective clinical investigation.

Responses to the survey questions varied considerably by specialty. Differences in opinion between specialties were significant for estimated case volume, preferred imaging, preferred treatment, and the management of asymptomatic lesions. These differences likely reflect standards of training within each field, clinical perspectives, experience, and philosophies within individual disciplines. It is not surprising that trauma surgeons see a large volume of TCVI cases and that CTA is their preferred method of imaging, since CT is currently widely used for imaging of trauma patients. Similarly, the observation that the majority (56.9%) of vascular surgeons prefer anticoagulation for treatment - more than any other specialty - may parallel practice guidelines for the treatment of other problems commonly encountered by vascular surgeons, such as peripheral arterial disease [50]. It is less clear why neurosurgeons, trauma surgeons, and general surgeons are more likely to use endovascular techniques to treat clinically silent TCVI lesions than vascular surgeons, neurologists, and interventional radiologists. The care of TCVI patients, particularly those with polytrauma, does typically involve the participation of multiple specialists. The large practice variation found by this survey highlights the utility of involving multiple specialties in future clinical trials of TCVI, and to include multiple specialties in the formulation of future practice guidelines.

Limitations of this study include the modest overall response rate and the variability in the numbers of respondents representing the different medical disciplines. However, as mentioned above, the respondents to this survey may represent a significant proportion of clinicians who actively participate in the management of TCVI in the United States. Another limitation concerns the restricted format of this survey. This single-page six-question format, without a large number of answer options for each question and without space to type out comments, was intended to keep the email survey brief to maximize recipient participation. In the view of some of the recipients of this survey, however, the brevity of the survey over-simplified the issues associated with TCVI management. The survey was meant to focus on the core questions without taxing the respondents' time and effort to an unreasonable degree.

## Conclusions

The results of this survey show that there is poor agreement on the management of patients with TCVI, from the method of imaging to medical and endovascular treatment and the handling of patients with asymptomatic lesions. These differing views reflect the absence of randomized trial data and well-defined treatment algorithms. Practice differences between medical disciplines underscores the need for and the value of multidisciplinary clinical trials and guidelines.

## Competing interests

The authors declare that they have no competing interests (political, personal, religious, ideological, academic, intellectual, commercial or any other) in relation to this manuscript.

## Authors' contributions

MRH participated in and contributed to all phases of the study. JAW participated in and contributed to all phases of the study. YSP, SMT, LPC, and BCW participated in designing, organizing, and implementing the survey. JR did the statistical analysis. All authors read and approved the final manuscript.
